# Endothelial Dysfunction in Fabry Disease Is Related to Glycocalyx Degradation

**DOI:** 10.3389/fimmu.2021.789142

**Published:** 2021-11-30

**Authors:** Solvey Pollmann, David Scharnetzki, Dominique Manikowski, Malte Lenders, Eva Brand

**Affiliations:** ^1^ Department of Internal Medicine D, and Interdisciplinary Fabry Center (IFAZ), University Hospital Muenster, Muenster, Germany; ^2^ Institute of Physiological Chemistry and Pathobiochemistry and Cells-in-Motion Cluster of Excellence (EXC1003-CiM), University of Muenster, Muenster, Germany

**Keywords:** Fabry disease (FD), endothelial dysfunction, heparanase, angiopoietin-1-receptor, globotriaosylsphingosine (lyso-Gb_3_), NF-κB

## Abstract

Fabry disease (FD) is an X-linked multisystemic lysosomal storage disease due to a deficiency of α-galactosidase A (*GLA*/AGAL). Progressive cellular accumulation of the AGAL substrate globotriaosylceramide (Gb_3_) leads to endothelial dysfunction. Here, we analyzed endothelial function *in vivo* and *in vitro* in an AGAL-deficient genetic background to identify the processes underlying this small vessel disease. Arterial stiffness and endothelial function was prospectively measured in five males carrying *GLA* variants (control) and 22 FD patients under therapy. AGAL-deficient endothelial cells (EA.hy926) and monocytes (THP1) were used to analyze endothelial glycocalyx structure, function, and underlying inflammatory signals. Glycocalyx thickness and small vessel function improved significantly over time (p<0.05) in patients treated with enzyme replacement therapy (ERT, n=16) and chaperones (n=6). AGAL-deficient endothelial cells showed reduced glycocalyx and increased monocyte adhesion (p<0.05). In addition, increased expression of angiopoietin-2, heparanase and NF-κB was detected (all p<0.05). Incubation of wild-type endothelial cells with pathological globotriaosylsphingosine concentrations resulted in comparable findings. Treatment of AGAL-deficient cells with recombinant AGAL (p<0.01), heparin (p<0.01), anti-inflammatory (p<0.001) and antioxidant drugs (p<0.05), and a specific inhibitor (razuprotafib) of angiopoietin-1 receptor (Tie2) (p<0.05) improved glycocalyx structure and endothelial function *in vitro*. We conclude that chronic inflammation, including the release of heparanases, appears to be responsible for the degradation of the endothelial glycocalyx and may explain the endothelial dysfunction in FD. This process is partially reversible by FD-specific and anti-inflammatory treatment, such as targeted protective Tie2 treatment.

## Introduction

Fabry disease (FD; Online Mendelian Inheritance in Man [OMIM] no. 301500) is an X-linked inherited disorder caused by a lack of the lysosomal enzyme α-galactosidase A (AGAL; EC 3.2.1.22) activity. The enzymatic deficiency leads to a lysosomal accumulation of the AGAL substrate globotriaosylceramide (Gb_3_) in most cell types of affected patients, including cardiac, renal, neuronal and vascular cells. The progressive accumulation results in a multisystemic disorder with early cerebrovascular events, heart failure, cardiac arrhythmia, and end-stage renal disease with a mean reduction of lifespan by 10 to 15 years (1). FD is treatable with enzyme replacement therapy (ERT) and chaperone therapy (migalastat) (2).

FD belongs to the so called “small vessel diseases”, affecting the microvasculature rather than the larger vessels. Several studies pointed out that Gb_3_ and the soluble globotriaosylsphingosine (lyso-Gb_3_) play an important role in endothelial dysfunction in FD mainly in the microvasculature (3, 4). The vascular endothelium acts as a selective permeable barrier between the blood and extravascular tissues. At the luminal side, vascular endothelial cells are covered by a functional structure of negatively charged proteoglycans, glycoproteins, and glycosaminoglycans (GAG) forming the endothelial glycocalyx (5–7). The glycocalyx thickness varies between ~0.2 and 10 µm depending on vessel bed or type (8, 9) and results from a balance between synthesis and shedding of sugar molecules and scaffold proteins. An important regulator of these processes is inflammation (7, 10, 11). In general, a thicker glycocalyx promotes vasculoprotective functions, whereas a degradation of the endothelial glycocalyx barrier results in an increased permeability for macromolecules and an increased leukocyte and platelet adhesion to the endothelium (12–14).

Since *in vivo* data on endothelial function in FD patients are scarce, the aim of our prospective study was to determine endothelial parameters including glycocalyx thickness and microvascular function as well as pulse wave velocity as an indicator of arterial stiffness in male patients with FD. Therefore, glycocalyx thickness and pulse wave velocity were repeatedly measured in 5 untreated controls and 22 treated males with FD including ERT and migalastat. To assess mechanisms involved in endothelial glycocalyx degradation, an FD cell model was established to analyze the effect of different treatment strategies including FD-specific therapy (ERT), antioxidants, non-steroidal anti-inflammatory drugs, steroids, anti-thrombotic as well as potential novel therapeutic approaches.

## Material and Methods

### Patients

For this prospective study, 27 male patients were recruited at the Interdisciplinary Fabry Disease Center (IFAZ) from the University Hospital Muenster. All analyses were performed after approval of the Medical Association of Westphalia-Lippe and the Ethical Committee of the Medical Faculty of the University of Muenster (project-nos.: 2011-347-f; 2011-186-f). Patients gave written informed consent for data analysis and publication. All investigations conformed to the principles outlined in the Declaration of Helsinki.

Blood pressure was measured in a quiet room and sitting position after 5 min of rest with a Mobil-O-Graph device (IEM, Stolberg, Germany), according to current guidelines (15–17). Pulse wave velocity (PWV) measurements were performed according in a quiet room after a 15 min rest in a sitting position by two well trained operators (18, 19). This device is validated according to the British Hypertension Society and European Society of Hypertension recommendations (15, 16, 20, 21). Cuffs were applied at the left upper arms in a sitting position. Microvascular imaging was performed in a supine position in a quiet room by two experienced operators (baseline and follow-up) as reported previously (22). To visualize the sublingual microvasculature, a SDF camera (CapiScope HVCS; KK Technology, Honiton, UK) was used to document at least 10 areas close to the frenulum, as described previously (22) and according to recent guidelines (23). Image acquisition and analysis were performed using the GlycoCheck software (Microvascular Health Solutions, Salt Lake City, UT). Three consecutive sets of measurements were performed for every patient, and individual mean values were calculated for analysis. Vessel density was calculated according to previous reports (22, 24). Patients’ mean PWV, augmentation indices, glycocalyx thickness, perfused boundary region (PBR5-25) and red blood cell (RBC) filling were determined out of three consecutive measurements.

Anti-drug antibodies (ADAs) were determined using the methodology previously described by Lenders and colleagues (25, 26).

### Cell Culture

Human vascular endothelial cell line EA.hy926 and CRISPR/Cas9-mediated AGAL-deficient EA.hy926 (EA.hy926-KO) generated and described previously (27) were cultivated in Dulbecco’s Modified Eagle Medium (DMEM; Thermo Fisher Scientific) containing 10% fetal calf serum (FCS, Sigma-Aldrich, St. Louis, Missouri, USA), 100 U/ml penicillin, 100 ng/ml streptomycin, and 2 mM L-glutamine (all Capricorn Scientific, Ebsdorfergrund, Germany). THP1 monocytes were cultivated in Roswell Park Memorial Institute 1640 (RPMI; Thermo Fisher Scientific) containing 10% FCS, 100 U/ml penicillin, 100 ng/mL streptomycin, 2 mM/ml L-glutamine, and 1% MEM non-essential amino acid solution (Sigma-Aldrich). CRISPR/Cas9-mediated AGAL knockout in THP1 (THP1-KO) cells was performed as previously described (28). The AGAL knockout resulted in less than 2% of wild-type AGAL activity in both cell lines (EA.hy926-KO and THP1-KO), representing a classical FD phenotype (**Supplemental Figure 1**).

### Western Blots

For nuclear detection of NF-κB 2 × 10^5^ cells/ml were seeded for 3 days and stimulated as stated. In short, cells were harvested in PBS and resuspended in buffer A (1 mM DTT; 10 mM KCl; 10 mM HEPES, pH 7.9; 1.5 mM MgCl2; protease inhibitor cocktail). After 15 min incubation on ice, 10% NP40 was added and centrifuged at 5,000 g for 5 min. Subsequently pellets were resuspended in buffer B (1.5 mM MgCl2; 1 mM DTT; 420 mM NaCl; 0.5 mM PMSF; 0.2 mM EDTA; 20 mM HEPES, pH 7.9; 25% (v/v) glycerol) and incubated for 30 min under frequent vortexing. After a final centrifugation step at 14,000 rpm, the nuclear faction (supernatant) was obtained. 20 µl of the nuclear fraction were separated on a 10% SDS gel. Subsequently, the samples were blotted onto a PVDF membrane and blocked overnight in 5% semi-skimmed milk powder (Roth, Karlsruhe, Germany) in Tris-buffered saline supplemented with 0.1% Tween 20 (TBST; AppliChem, Darmstadt, Germany). Anti-p105/50 (Cell Signaling Technology, Danvers, Massachusetts, USA; 13586, 1:3,000) was used to detect NF-κB and visualized with a secondary horseradish peroxidase-conjugated goat anti-rabbit antibody (Merck Millipore, Burlington, Massachusetts, USA, 12-348, 1:10,000). Loading control was performed with monoclonal PCNA (Santa Cruz Biothechnology, Dallas, Texas USA; sc-56, 1:500).

To detect angiopoietin-2 (Angpt-2) expression, 2 × 10^5^ cells/ml were seeded onto 6 wells. Intracellular angiopoietin 2 expression was detected using an anti-angiopoetin-2 antibody (Abcam, Cambridge, UK; ab155106) combined with a HRP-coupled secondary antibody (anti-rabbit IgG [Merck; no: 12-348]).

### *In Vitro* Glycocalyx Detection and Fluorescence Microscopy

Fucose stainings were performed to determine the glycocalyx on the cellular surface. Stainings were quantified by a plate reader and by flow cytometric analyses and visualized by fluorescence microscopy. For flow cytometric analyses, EA.hy926 and AGAL-deficient EA.hy926 cells were seeded at a density of 1.5 × 10^5^ cells/ml on 48-well plates for 72 h. For fluorescence microscopy, 2 × 10^5^ cells/ml of cells were seeded on glass cover slips in 24-well plates for 96 h until confluence. To determine the glycocalyx by a plate reader, 2 × 10^5^ cells/ml were seeded on a 96-well plate (Greiner Bio One, Frickenhausen, Germany; 655090). Treatments of AGAL-deficient EA.hy926 cells were carried out at seeding for indicated times. Cells were treated with 20 µg/ml agalsidase-beta (Sanofi-Genzyme, Amsterdam, Netherlands; 11541667), 0.4 U/ml heparin (Sigma-Aldrich), 10 µM AKB-9778 (razuprotafib; MED Chem Express, Monmouth Junction, New Jersey, USA) for 48 h. Additionally, for long-term treatment AGAL-deficient EA.hy926 cells were cultivated for four to five weeks with 0.4 U/ml heparin or 10 µM AKB-9778 before subsequent experiments. Treatments with 20 nM dexamethasone (dexa; Sigma-Aldrich, D4902), 60 µM N-acetylcysteine (NAC; Sigma-Aldrich; A9165), 100 µM acetylsalicylic acid (ASA; Sigma- Aldrich; A5376) were performed for 96 h. To quantify the amount of glycocalyx by plate reader analyses, cells were co-stained with 5 µg/ml UEA-1-fluorescein isothiocyanate (UEA-1-FITC; Thermo Fisher Scientific, Eugene, Oregon, USA; L32476) and 5 µg/ml Hoechst (Thermo Fisher Scientific, H1399) for 20 min in HBSS at 37°C. After removing the staining solution, 100 µl HBSS per well were added and fluorescence was measured at 459 nm and 515 nm for UEA-1-FITC and at 350 nm and 461 nm for Hoechst (Tecan, Infinite M200, Maennedorf, Switzerland). The fluorescence intensity of UEA-1-FITC was normalized against Hoechst.

To visualize the glycocalyx by fluorescence microscopy, cells cultivated on glass cover slips were washed with HBSS followed by a staining with 5 µg/ml UEA-1 FITC for 20 min at 37°C in HBSS. For microscopy-based NF-κB detection, cells were seeded on glass cover slips. After stimulation, cells were fixed with 4% paraformaldehyde and permeabilized with 0.2% Triton X-100 (Carl Roth, Karlsruhe, Germany; 3051.2) in PBS. Samples were incubated overnight with primary rabbit anti-p105/50 antibody (Cell Signaling Technology; 1:1,000). The secondary antibody was an Alexa594-labeled anti-rabbit IgG (Thermo Fisher Scientific; A11012, 1:2000). Fluorescence microscopy was performed using an Axio Observer Z1 fluorescence microscope (Carl Zeiss, Oberkochen, Germany) with a 63-fold oil objective. Images were processed using the Axiovision software 2.3 (Carl Zeiss).

Cell adherence of monocytes on endothelial cell was additionally visualized by fluorescence microscopy. After removal of non-adherent THP1 monocytes cells were fixed with 4% para-formaldehyde solved in PBS. After permeabilization with 0.2% Triton X100 in PBS, cells were incubated with phalloidin-FITC (Enzo Life Sciences, ALX-350-268-MC01; working concentration: 0.4 µg/ml) and mouse anti-human CD32 allophycocyanin (CD32-APC; BD Bioscience, 559769; working concentration 1:5) in 0.5% bovine serum albumin (BSA) in PBS for 30 min at room temperature to stain filamentous actin (F-actin) and CD32-APC. Fluorescence microscopy was performed using Axio Observer Z1 fluorescence microscope (Carl Zeiss, Oberkochen, Germany) with a 40-fold oil objective. Images were processed using the Axiovision software 2.3 (Carl Zeiss) and ImageJ (v1.51n) (29).

For flow cytometry analyses, EA.hy926 cells were dissolved with enzyme-free cell dissociation buffer (Life Technologies, Bleiswijk, Netherlands; 13151-014) and washed with PBS after indicated cultivation times. After staining of fucose with 5 µg/ml UEA-1-FITC (Thermo Fisher Scientific) in PBS for 20 min at room temperature, cells were washed again. Finally, cells were analyzed using a fluorescence-activated cell sorting (FACS) Canto II flow cytometer (BD Bioscience, Franklin Lakes, New Jersey, USA) and the data were evaluated using BD FACSDiva software (BD Bioscience) and FlowJo (FlowJo LLC, Ashland, Oregon, USA).

### Heparanase 1 Expression

To quantify heparanase 1 expression *via* quantitative real-time-PCR in EA.hy926 and AGAL-deficient EA.hy.926-KO cells, these were seeded in a cell density of 1.5 x 10^5^ cells in 6-well plates and grown for 48 h. Wild-type EA.hy926 cells were stimulated with TNFα (10 ng/ml) for the last 24 h as positive controls. RNA was extracted using the NucleoSpin RNA extraction kit (740955, Macherey-Nagel, Dueren, Germany) according to manufacturer’s protocol. mRNAs were reverse-transcribed into cDNA by using cDNA synthesis kit (Thermofischer, K1612), 1 µg total RNA and Oligo-dT primers according to manufacturer’s instructions. mRNA levels were determined by using QuantiNova SYBR Green PCR kit (QIagen, 208044) on a BioRad CFX384 Real-Time-System C1000 Touch Thermal Cycler. β-actin was used as a reference mRNA and relative expression was calculated with the ΔΔct method. The following primer were used: hACTP sense 5’-CGT GTG GAT CGG CGG CTC-3´, hACTB antisense 5´-TAG GTT TTG TCA AGA AAG GGT G-3´, hHPSE sense 5´-GCA CAA ACA CTG ACA ATC CAA GG-3´, hHPSE antisense 5´-AGG TCC CAA AGG TCT TAG AAG GT-3´.

### Monocyte Adhesion Assays

To assess monocyte adhesion on endothelial cell layers, wild-type EA.hy926 cells were incubated with wild-type THP1 monocytes and AGAL-deficient EA.hy926 cells with AGAL-deficient THP1 monocytes to simulate a healthy or FD situation. Endothelial cells were seeded at a density of 1.5 × 10^5^ cells/ml on glass cover slips in 24-well plates and cultured for 72 h or 96 h until confluence. Treatments of AGAL-deficient EA.hy926 cells were carried out at seeding for indicated times. Treatments with 20 µg/ml agalsidase-beta (Sanofi-Genzyme), 1 µM atorvastatin (PZ0001), 1 µM mevastatin (M2537), or 1 µM simvastatin (S6196) (all Sigma-Aldrich) were conducted for 96 h including a medium change 48 h after seeding. Treatment with 20 nM dexamethasone (Sigma-Aldrich; D4902), 60 µM NAC (Sigma-Aldrich), 100 µM acetylsalicylic acid (ASA; Sigma-Aldrich; A5376), 10 µM AKB-9778 (MEDChemExpress), or 0.4 U/ml heparin (Sigma-Aldrich) was performed for 72 h. Furthermore, AGAL-deficient EA.hy926 cells were treated for four to five weeks with 10 µM AKB-9778 or 0.4 U/ml heparin before appropriate experiments. After indicated treatment time 1.25 × 10^5^ THP1 or AGAL-deficient THP1 monocytes in DMEM containing 10% FCS, 100 U/ml penicillin, 100 ng/ml streptomycin, and 2 mM L-glutamine were added to the endothelial monolayers. Cells were incubated for 30 min at 37°C before non-adherent monocytes were removed by gently transferring the cover slips twice to new wells containing fresh PBS.

To quantify the amount of adherent THP1 monocytes (wild-type or AGAL-deficient) flow cytometry analyses were performed. Therefore, adherent THP1 monocytes and EA.hy926 cells were dissociated using enzyme-free cell dissociation buffer (Life Technologies, Bleiswijk, Netherlands; 13151-014). Subsequently, cells were washed with 0.5% BSA in PBS and incubated with mouse anti-human CD32 APC (BD Bioscience) in 0.5% BSA in PBS for 30 min at 4°C. After a final washing step with 0.5% BSA in PBS, stained monocytes were detected using a FACS Canto II flow cytometer. Data were analyzed using BD FACSDiva software and FlowJo.

### Quantification of Sulfated Glycosaminoglycan in Urine

To quantify the amount of sulfated glycosaminoglycans (GAG) in the urine dimethylmethylene blue (DMMB) assays modified from Sun and colleagues (30) were performed. Urine samples were thawed on ice. Chondroitin 4 sulfate (Sigma-Aldrich; 27042) served as standard with a calibration curve ranged from 0 to 50 µg/ml solved in H_2_O. 25 µl of the urine samples per 96-well were incubated with 200 µl of DMMB solution (for 1 liter: 16 mg DMMB (Sigma-Aldrich; 341088), 3.05 g glycine, 1.6 g sodium chloride, 544 µl acetic acid, pH 4). The GAG concentration of each urine sample was calculated from the chondroitin 4 sulfate standard curve and then normalized to urine creatinine concentration. Samples out of range were repeated in serial dilution.

### Statistics

All experiments were performed at least three times. Continuous variables were expressed as mean with standard deviation (SD). Unpaired two-tailed Student’s t-test or one-way analysis of variance (ANOVA) with correction for multiple testing was used for statistical analysis where appropriate. Analyses and visualizations were performed with GraphPad PRISM v8.0 software (GraphPad Software Inc., La Jolla, CA, USA).

## Results

### Pulse Wave Velocity, Augmentation Index and Glycocalyx Measures in FD Patients

**Figure 1:**
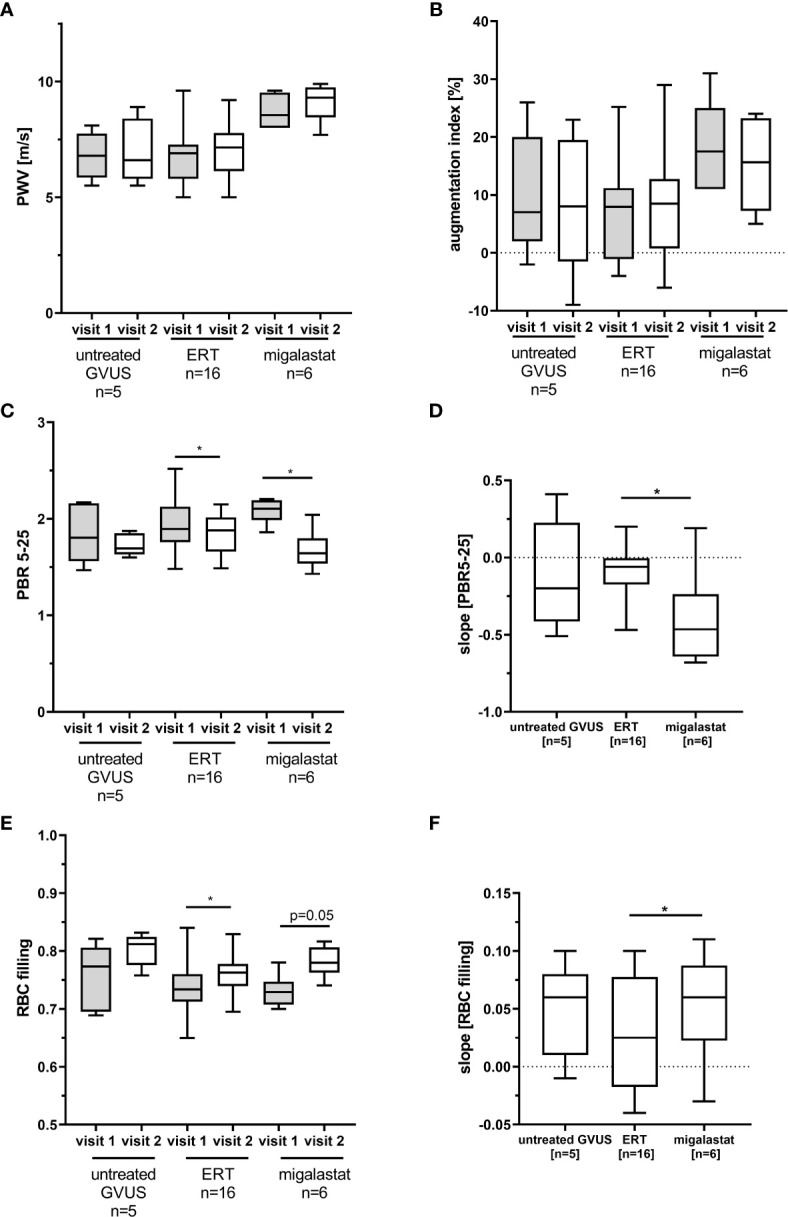
Pulse wave velocity and glycocalyx measures in patients with FD. **(A)** Pulse wave velocity (PWV) and **(B)** augmentation index measures in FD patients and control patients with genetic variants of unknown significance (GVUS). **(C)** Perfused boundary region (PBR) in small vessels (5 to 25 µm in diameter). **(D)** Changes for PBR in one year. **(E)** Red blood cell (RBC) filling in small vessels. **(F)** Changes for RBC filling in one year. ERT, enzyme replacement therapy. *p < 0.05 determined by Kruskal-Wallis test or One-Way ANOVA. Visit 1: first visit, study start, Visit 2: follow-up visit.

To analyze potential vascular and endothelial damage in patients with FD, we performed pulse wave velocity (PWV), augmentation index and glycocalyx measures in ERT- (n=16) and migalastat-treated (n=6) male patients at two consecutive follow-up visits within 12 months (visit T1 and visit T2). Untreated males (n=5) carrying genetic variants of unknown significance (GVUS) within the *GLA* gene (p.S126G and p.A143T) with enzymatic AGAL activities within the reference range, normal lyso-Gb_3_ values, lacking FD-typical manifestations and symptoms served as controls. Baseline characteristics are presented in **Table 1**. Patients under ERT were not treatment-naïve and treated with ERT for a mean duration of 102 ± 64 months at first (T1) visit. In contrast, two patients (33.3%) treated with migalastat were treatment-naïve at T1 and the remaining four patients (66.7%) received ERT for 32 ± 29 months before being switched to migalastat (**Table 1**). Migalastat-treated FD patients were significantly older than ERT-treated patients (p<0.05; **Table 1**). No significant differences for eGFR were observed between the three groups. The interventricular septum thickness was significantly increased in migalastat-treated patients compared to untreated controls and ERT-treated patients (**Table 1**). This cardiac involvement in migalastat-treated patients might be due to the increased age and higher central systolic blood pressure (cSBP) as well as the high frequency of the cardiac variant p.N215S (66.7%) in this group (**Table 1**). PWV and augmentation index did not differ between the three groups and were stable over time (**Figures 1A, B**). The perfused boundary region (PBR) as an inverse marker for glycocalyx thickness in microvascular vessels remained stable in untreated control patients, but decreased significantly in ERT- and migalastat-treated patients, demonstrating an increase in glycocalyx thickness over time (**Figure 1C**). Increase of glycocalyx was more prominent in migalastat-treated patients compared to those under ERT **(** **Figure 1D**). Red blood cell (RBC) filling also improved over time in treated patients (**Figure 1E**) and was more prominent in patients receiving migalastat, too (**Figure 1F**).

**Table 1:** Characteristics of the study cohort at first GlycoCheck and PWV measurement (T1).

measures	GVUS (n = 5)	ERT-treated (n = 16)	Migalastat-treated (n = 6)	p-value
**Age, years**	45 ± 10	45 ± 13	60 ± 8^†^	0.0338
Follow-up duration, months*	12 ± 2	13 ± 2	14 ± 9	0.8160
**Pretreated with ERT, months**	0	102 ± 64	32 ± 29	0.0185
**ADAs, %**	0 (0.0)	8 (50.0)	0 (0.0)	0.0201
**Agalsidase-alfa, %**	0 (0.0)	9 (56.2)	0 (0.0)	n.a.
**Creatinine, mg/ml**	1.08	1.26	1.08	0.4420
	[0.90 to 1.29]	[0.77 to 9.50]	[0.75 to 1.66]	
**eGFR, ml/min/1.73 m²**	82.7 ± 19.8	65.7 ± 37.4	73.2 ± 17.5	0.5729
**SBP, mmHg**	125.0 ± 7.0	120.3 ± 10.0	131.5 ± 9.3	0.0612
**DBP, mmHg**	83.6 ± 9.9	78.9 ± 11.2	88.8 ± 8.3	0.1535
**cSBP, mmHg**	117.4 ± 6.9	110.3 ± 9.2	120.8 ± 7.8^†^	0.0375
**Interventricular septum thickness, mm**	10.0	13.8	21.5	0.0307
	[10.0 to 13.0]	[7.7 to 27.0]	[10.2 to 30.0]^‡^	
**Mutations included**	2x p.S126G, 3x p.A143T	1x p.L294S,	4x p.N215S,	
		1x p.H225D,	1x p.I242V,	
		1x p.L45P,	1x p.R259P	
		1x c.1090-1103del,		
		1x p.R301Q,		
		3x p.R220X,		
		1x IVS2+1G>A,		
		1x p.W162G,		
		1x c.801+3A>T,		
		1x p.R342Q,		
		1x p.C202Y,		
		1x p.S247P,		
		1x p.I242fsx,		
		1x p.W349X		

ADA, anti-drug antibody; cSBP, central systolic blood pressure; DBP, diastolic blood pressure; eGFR, estimated glomerular filtration rate; GVUS, genetic variants of unknown significance; SBP, systolic blood pressure; ERT, enzyme replacement therapy; n.a., not applicable. *between first (T1) and follow-up (T2) visit. ^†^ERT- vs Migalastat-treated group: p<0.05. ^‡^GVUS- vs Migalastat-treated group: p<0.05.

### *In Vitro* Characterization of the Endothelial Glycocalyx in Fabry Disease

**Figure 2:**
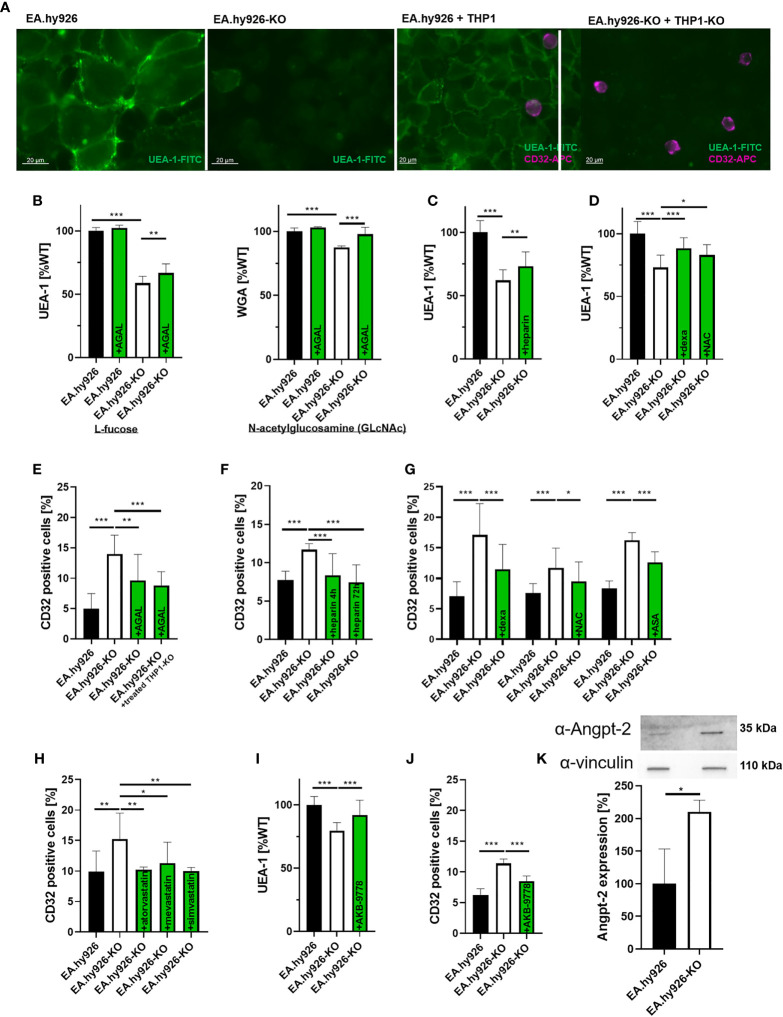
*In vitro* characterization of the endothelial glycocalyx in Fabry disease. **(A)** L-fucose stainings (green) of wild-type and AGAL-deficient EA.hy926-KO cells (left) and detection of adherent monocytes with CD32-APC (violet) antibodies (right). **(B)** L-fucose (UEA-1) and N-acetylglucosamine (WGA) stainings of wild-type and AGAL-deficient EA.hy926 cells revealed less glycocalyx in AGAL-deficient cells, which could be improved by 20 mg/ml agalsidase-beta (AGAL) treatment for 48 hours. **(C)** Heparin treatment (0.4 U/ml for 48 h), **(D)** dexamethasone (dexa, 60 µM) and N-acetylcysteine (NAC) (20 nM) treatment of AGAL-deficient cells protected glycocalyx. **(E)** THP1 adhesion was increased in an AGAL-deficient background and decreased after treatment with 20 mg/ml agalsidase-beta for 96 h. **(F)** Heparin (0.4 U/ml) and **(G)** dexamethasone (60 µM), NAC (20 nM), and acetylsalicyl acid (ASA, 100 µM) treatment for 72 h decreased monocyte adhesion on AGAL-deficient EA.hy926 cells. **(H)** Statins (all 1 µM for 96 h) reduced monocyte adhesion in an AGAL-deficient background to wild-type level. Treatment with the Tie2 activator AKB-9778 resulted in **(I)** increased glycocalyx thickness and **(J)** reduced monocyte adhesion in an AGAL-deficient background. **(K)** Angiopoietin-2 (Angpt-2) expression was increased in AGAL-deficient endothelial EA.hy926-KO cells. Experiments in **(B, D, I)** were quantified in a plate reader. Experiments in **(E, H, J)** were quantified by flow cytometry. AGAL, α-galactosidase A, UEA-1, Ulex europaeus agglutinin I, WGA, Wheat germ agglutinin. *p < 0.05, **p < 0.01, **p < 0.001 determined by Kruskal-Wallis test or One-Way ANOVA **(B–J)** or students t test **(K)**.

To further assess an impact of AGAL-deficiency on endothelial cell function, we used a stable AGAL-deficient endothelial cell line (EA.hy926-KO) (27) to analyze the glycocalyx composition and its function. AGAL-deficient cells demonstrated significantly less L-fucose and N-acetylglucosamine (GlcNAc) on their cellular surface (p<0.001, **Figures 2A, B**), which are major components of the glycocalyx. In line with the FD patient-derived data, treatment of EA.hy926-KO cells with recombinant AGAL (20 mg/ml) increased L-fucose and GlcNAc on AGAL-deficient cells by 8.0% and 10.0%, respectively (both p<0.001, **Figure 2B**). An important process for glycocalyx degradation is the expression and release of heparanases due to an inflammatory stimulus. To test whether heparanases might be involved in glycocalyx degradation in our model system, we analyzed heparanase I expression *via* qPCR. Indeed AGAL-deficient EA-hy926-KO cells showed an increased (6-fold) heparanase I expression compared to wild-type (**Supplemental Figure 2A**). Consequently, AGAL-deficient cells were treated with heparin, which is known to inhibit heparanase activity to analyze whether heparanases might be responsible for the observed glycocalyx reduction in FD. Indeed, treatment of AGAL-deficient cells with heparin for 48 h resulted in an increase of the glycocalyx by 11% (p=0.0013; **Figure 2C**). In addition, treatment with anti-inflammatory drugs (NSAIDs, dexamethasone) as well as N-acetylcysteine (NAC) as an antioxidant for 72 h showed comparable effects (p<0.001 and p=0.0154, respectively; **Figure 2D**). To further demonstrate that secreted enzymes (i.e. heparanases) are involved in the observed glycocalyx degradation, wild-type EA.hy926 cells were treated with supernatants from AGAL-deficient EA.hy926 cells (**Supplemental Figure 2B**). Treatment for 30 minutes with supernatants from AGAL-deficient cells resulted in a significant decrease of glycocalyx (p=0.0007), which could be reversed by a co-incubation with heparin (p=0.0225; **Supplemental Figure 2B**).

Since a reduction of the glycocalyx facilitates monocyte adhesion and invasion, we additionally analyzed monocyte adhesion on endothelial cells. To simulate a comprehensive AGAL-deficient background comparable to a situation *in vivo*, we generated CRISPR/Cas9-mediated AGAL-deficient THP1 monocytes based on a previously established method (28). Of note, only THP1 monocytes were positive for CD32 expression and showed no differences between wild-type and AGAL-deficient cells, allowing a proper quantification by flow cytometry analysis (**Supplemental Figure 3A**). In an AGAL-deficient background (i.e. AGAL-deficient EA.hy926 cells combined with appropriate THP1-KO monocytes), monocyte adhesion was increased compared to wild-type (p<0.0001; **Figures 2A, E**). Furthermore, the stimulation of wild-type as well as AGAL-deficient endothelial cells with TNFα demonstrated that both cell lines were yet able to react on an external inflammatory stimulus (**Supplemental Figure 3B**). Treatment of AGAL-deficient EA.hy926 cells with recombinant AGAL for 96 h significantly reduced monocyte adhesion (p=0.0045; **Figure 2E**). AGAL-treatment of both AGAL-deficient cell lines (EA.hy926-KO and THP1-KO) did not result in a more prominent monocyte adhesion, suggesting that the endothelial cells are responsible for the observed increased adhesion, rather than the monocytes (p=0.9348; **Figure 2E**). Treatment with heparin for 4 or 72 h resulted in a decreased monocyte adhesion to AGAL-deficient EA.hy926 cells (both p<0.001; **Figure 2F**). Vice versa, the incubation of wild-type EA.hy926 cells with conditioned supernatants of AGAL-deficient cells resulted in an increased (p=0.0005), heparin-treatment-reversible monocyte adhesion (p=0.0005) on wild-type EA.hy926 cells, also pointing towards an involvement of secreted heparanases (**Supplemental Figure 2C**). Incubation with anti-inflammatory drugs (dexamethasone and acetylsalicylic acid [ASA]) and the antioxidant NAC all resulted in a decreased monocyte adhesion in an AGAL-deficient background, too (**Figure 2G**). However, monocyte adhesion is not only mediated by glycocalyx thickness, but also by monocyte-adhesion receptors. Thus, we analyzed if statins, which are known to reduce monocyte adhesion by for example CD11b inhibition (31), also decrease cell adhesion in a FD background. Treatment with atorvastatin, mevastatin, and simvastatin for 96 h reduced monocyte adhesion, significantly (all p<0.01; **Figure 2H**).

### Selective TIE2 Activation Prevents Glycocalyx Degradation and Restores Endothelial Function *In Vitro*

Recent studies demonstrated that heparanase release is mediated by angiopoietin-1-receptor (Tie2) signaling, in that Angpt-2 mediates the binding of vascular endothelial protein tyrosine phosphatase (VE-PTP) to Tie2, followed by a release of heparanases. Razuprotafib (AKB-9778) is a novel Tie2 activator preventing the binding of VE-PTP to Tie2 and thus the release of heparanases (32, 33). Consequently, we analyzed whether AKB-9778 also inhibits a heparanase release in an AGAL-deficient background. Indeed, treatment of AGAL-deficient EA.hy926 cells with 10 µM AKB-9778 resulted in an increase of L-fucose (48 h) and a decreased monocyte adhesion (72h), too (both p<0.001; **Figures 2I, J**), indicating that Tie2 and Angpt-2 are also involved in the observed glycocalyx degradation in FD. An involvement of Angpt-2 was supported in that AGAL-deficient EA.hy926 cells revealed an increased Angpt-2 expression compared to wild-type (p=0.0272, **Figure 2K**).

### Lyso-Gb_3_ and AGAL-Deficiency Activate NF-κB-Mediated Glycocalyx Degradation in Endothelial Cells

**Figure 3:**
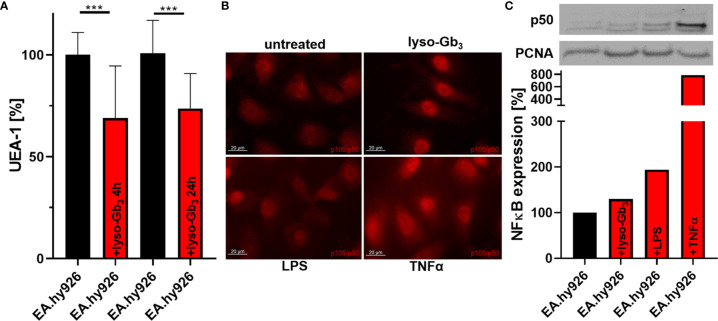
Lyso-Gb_3_ and AGAL-deficiency activate NF-κB-mediated glycocalyx degradation in endothelial cells. **(A)** Incubation of wild-type EA.hy926 cells with lyso-Gb_3_ (200 nM) led to degradation of the glycocalyx. **(B, C)** Treatment of wild-type EA.hy926 cells with pathologic lyso-Gb_3_ levels (200 nM), LPS (1 µg/ml in B and 100 ng/ml in **(C)** and TNFα (10 ng/ml) for 4 h results in increased nuclear NF-κB (p50 subunit) localization. Representative western blot of nuclear fractions and analysis from N=3 independent experiments. LPS, lipopolysaccharide; UEA-1, Ulex europaeus agglutinin I; lyso-Gb_3_, globotriaosylsphingosine. ***p < 0.001determind by students t test.

Previous studies demonstrated that lyso-Gb_3_ may trigger inflammatory signaling in cell culture (34). To analyze a potential direct effect of lyso-Gb_3_ on glycocalyx thickness, wild-type EA.hy926 cells were treated with a pathological lyso-Gb_3_ concentration of 200 nM. Interestingly, treatment for 4 or 24 h resulted in a decrease of glycocalyx (both p<0.001; **Figure 3A**), suggesting a rapid, lyso-Gb_3_-mediated heparanase release. Since AGAL-deficient EA.hy926 cells showed an increased NF-κB (p50) translocation (**Supplemental Figure 4**), we further analyzed whether pathologic lyso-Gb_3_ levels may activate nuclear translocation of NF-κB, too. Strikingly, treatment of wild-type EA.hy926 cells with 200 nM lyso-Gb_3_ for 4 h resulted in increased nuclear p50 signals determined by fluorescence microscopy (**Figure 3B**) and western blots (**Figure 3C**).

### Detection of Glycocalyx Degradation *In Vivo*

**Figure 4:**
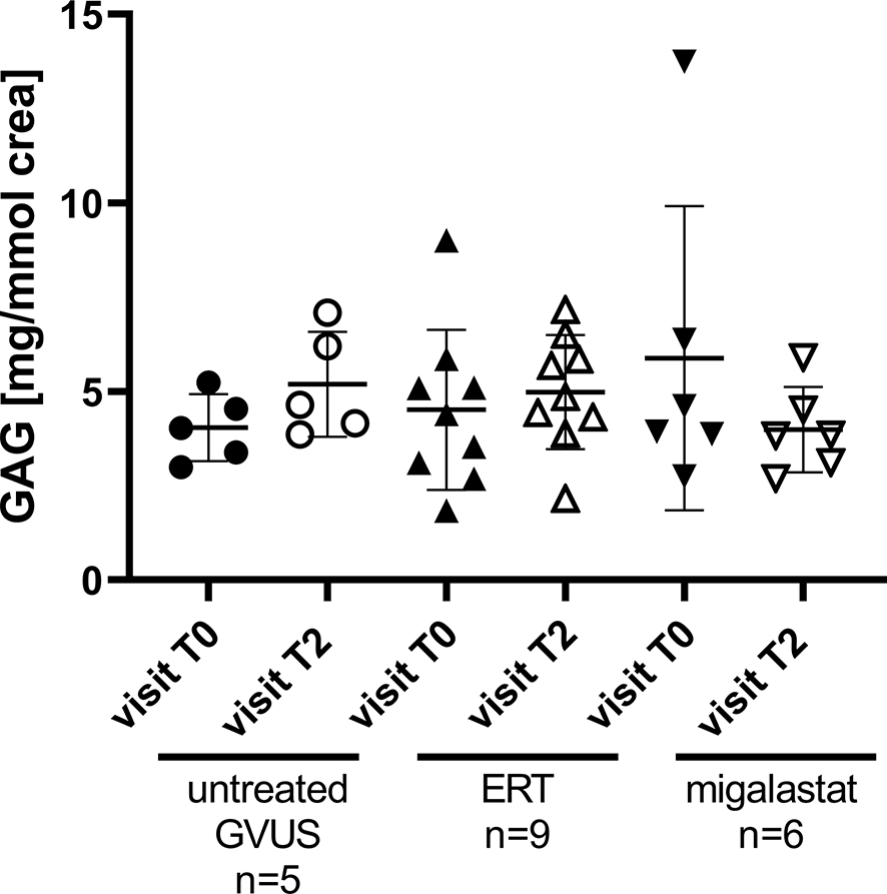
Urinary GAG content in patients with GVUS and FD patients after ERT or migalastat treatment. crea, urinary Creatinine; ERT, enzyme replacement therapy; GAG, glycosaminoglycans; GVUS, genetic variant of unknown significance. Analyzed by One-Way ANOVA. T0, first; treatment-naïve visit (retrospective); T2, last/current visit.

Our cell culture experiments pointed to an increased glycocalyx degradation by inflammatory processes due to FD. Recent studies demonstrated measurable changes in urinary GAG levels in patients suffering from sepsis. To analyze if a continuous degradation of the glycocalyx in FD patients under FD-specific treatment might also be detectable, we additionally measured GAGs in urine samples from a subset of treated patients at two time-points. At the first time-point (T0) all patients were naïve to any FD-specific therapy. The second measurements were performed in urine samples from the control group and ERT- or migalastat-treated patients from the last (current) visit (T2), which is the same visit as for glycocalyx measurements. Urinary GAG levels were in all groups and all time points comparable and thus constant (**Figure 4**).

## Discussion

Endothelial dysfunction is one of the main manifestations in FD and yet poorly understood. In the current work we analyzed the endothelial glycocalyx and its function *in vivo* and in cell culture. Our main results are as follows: 1) Glycocalyx thickness and RBC count in small vascular vessels in patients with FD improved after FD-specific treatment with ERT or migalastat *in vivo*. 2) In FD, glycocalyx degradation seems to be mediated by different inflammatory processes involving Angpt-2, potentially leading to the release of heparanases. 3) FD-specific therapy, anti-inflammatory drugs, heparin and specific Tie2 activation can reverse glycocalyx degradation.

The partial degradation of the endothelial glycocalyx by the release of heparanases is one of the first responses to an inflammatory stimulus within the endothelium. This mechanism may lead to a more exposed position of surface located receptors such as monocyte adhesion molecules and an increased endothelial leakiness, allowing pro-inflammatory cytokines and monocytes to invade through the endothelial barrier. However, persistent degradation of the glycocalyx results in a chronic inflammatory response, leading to a severe damage of the vascular endothelium. In this respect, as known from septic patients, the extent of glycocalyx degradation correlates with patients’ morbidity and mortality (35). Furthermore, endothelial glycocalyx is more reduced in mechanically vented patients with COVID-19 then in non-mechanically vented patients (36). FD belongs to the small vessel diseases and affected patients present with a chronically increased inflammatory status and endothelial dysfunction. As a consequence, patients suffer from FD-related vasculopathy (3). Our PWV measures demonstrated no differences between the patient groups and treatments, confirming the small vessel disease by revealing no obvious impairment of the large vessels. However, to the best of our knowledge, we are the first, demonstrating a reduced glycocalyx thickness in affected patients with FD *in vivo*. Under FD-specific treatment (including ERT and migalastat), glycocalyx and the red blood cell filling as a marker for the microvascular perfusion improved, pointing towards a reversible, FD-related mechanism of glycocalyx degradation.

Our cell culture experiments indicated increased release of heparanases as the cause of this effect. Direct inhibition of heparanases by heparin, the application of antioxidants and anti-inflammatory drugs and also recombinant AGAL prevented the glycocalyx degradation. Our data demonstrate the involvement of pro-inflammatory cytokines as well as (lyso)-Gb_3_ in this glycocalyx degradation. An improvement of the endothelial glycocalyx was associated with a decreased monocyte adhesion, highlighting the importance to protect the endothelial glycocalyx in patients at risk. Endothelial glycocalyx degradation is mediated by Angpt-2 (37) and thus by Tie2. A recent study demonstrated a protective effect of vasculotide (Vasomune therapeutics, Toronto, CA) on the glycocalyx, in that the release of heparanases was prevented by blocking Angpt-2 (38). To analyze whether a comparable effect might be achieved in FD, we used the compound AKB-9778 (razuprotafib, Aerpio), which is known to inhibit the binding of VE-PTP to Tie2, preventing subsequent heparanase release as well as to stabilizes general cell connectivity in the presence of Tie2 (33). The use of razuprotafib instead of vasculotide had the advantage that the complete pathologic mechanism could be blocked instead of inhibiting one specific downstream-located protein (Angpt-2). Strikingly, our data demonstrated a protected glycocalyx and decreased monocyte adhesion in cell culture after razuprotafib treatment, pointing towards an Angpt-2-dependent mechanism, which is VE-cadherin-independent (33). These data are relevant to FD since drugs targeting the Tie2 pathway, such as AKB9778, are currently being investigated for the treatment of retinal vascular disease caused by diabetic nephropathy (39–41), hypertension in patients with diabetes mellitus (42) as well as the acute respiratory distress syndrome due to COVID-19 infections (NCT04511650). Thus, these novel drugs could be very useful as future adjunctive therapy in FD. Unlike sepsis or other acute inflammation-triggered diseases, resulting in a decrease of glycocalyx thickness and also measurable higher urinary GAG levels in a short period of time, FD results probably rather in a chronic impairment and/or restructuring of the glycocalyx. Therefore, it may not be expected that urinary GAG levels will change significantly over time in these patients. Future work should analyze the composition of glycocalyx in patients with FD.

**Figure 5:**
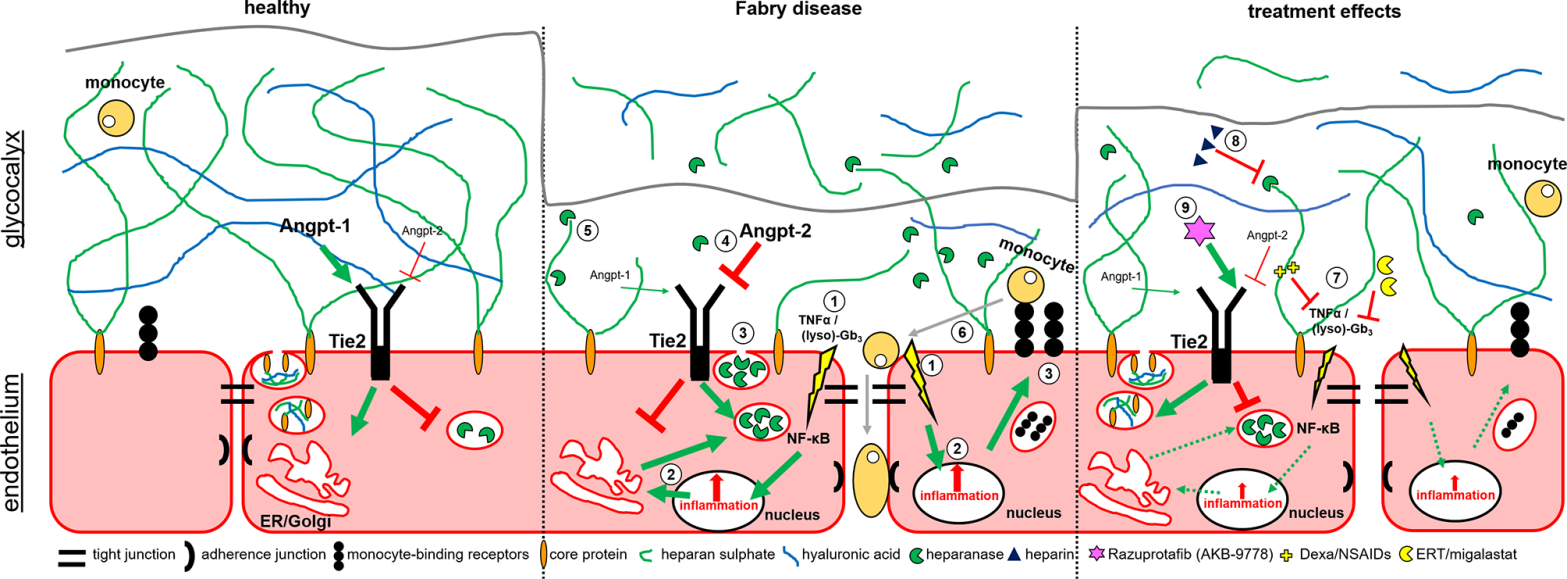
Potential mechanisms involved in glycocalyx damage and endothelial dysfunction in Fabry disease. Schematic overview of the endothelial cell layer in healthy (left), Fabry disease (middle) and treated Fabry disease (right). 1. Lyso-Gb_3_ and pro-inflammatory cytokines (TNFα) mediate NF-κB-mediated inflammatory signaling and gene expression. 2. Inflammatory response leads to an increased expression of heparanases and pro-inflammatory proteins such as monocyte-binding receptors. 3. Release of heparanases and presentation of monocyte-binding factors. 4. In addition, high Angpt-2 expression leads to a binding of Vascular Endothelial Protein Tyrosine Phosphatase (VE-PTP) to Tie2 and thus a direct release of heparanases. 5. Heparanases degrade the glycocalyx, leading to a release of free GAGs and hyaluronic acid (HA). 6. Damaged glycocalyx and increased expression of monocyte-binding receptors lead to an increased binding of monocytes to cell adhesion molecules with subsequent endothelial invasion. Inflammatory stimulus increases the expression of cell adhesion molecules on the cell surface. 7. FD-specific therapy (ERT, migalastat) or anti-inflammatory drugs (NSAIDs, dexamethasone) decrease the (lyso)-Gb_3_- and/or TNFα-mediated inflammatory stimuli. 8. Heparin inhibits heparanases, preventing glycocalyx degeneration. 9. Razuprotafib (AKB-9778) inhibits the binding of VE-PTP to TIE2 preventing subsequent heparanase release.

Based on our data, we here provide new insights in mechanisms involved in endothelial dysfunction in FD (**Figure 5**). In FD, lyso-Gb_3_ and pro-inflammatory cytokines (e.g. TNFα) activate NF-κB-mediated inflammatory signaling and target gene expression. The inflammatory response leads to an increased expression (and release) of heparanase and pro-inflammatory proteins such as monocyte-binding receptors. In addition, high Angpt-2 expression results in the binding of vascular endothelial protein tyrosine phosphatase (VE-PTP) to Tie2 and thus a direct release of heparanases. Damaged glycocalyx and increased expression of monocyte-binding receptors can lead to a facilitated binding of monocytes to cell adhesion molecules with subsequent endothelial invasion. Furthermore, the weakening of the endothelial barrier function may activate smooth muscle cells, which are also hypothesized to be involved in the vasculopathy of FD (3). FD-specific therapy (ERT, migalastat), and anti-inflammatory drugs (NSAIDs, dexamethasone) decrease the (lyso)-Gb_3_- or TNFα-mediated inflammatory stimulus. In addition, the treatment of AGAL-deficient EA.hy926 cells with NAC resulted in an increase of the glycocalyx and decreased monocyte adhesion, too. The observed effects might be due to the primary anti-oxidative function of NAC by replacing superoxide dismutase function, which proper function could be disturbed by the loss of interaction with the endothelial glycocalyx (43) and seems to be downregulated in FD patient-derived endothelial cells (44). Thus, a reduction of oxidative stress might result in a decreased pro-inflammatory signaling in FD. However, further studies are required to analyze the impact of oxidative stress in FD in more detail.

Heparin inhibits heparanases, thereby preventing glycocalyx degradation and razuprotafib (AKB-9778) directly inhibits the binding of VE-PTP to Tie2, preventing subsequent heparanase release.

We conclude that a chronic degradation of the endothelial glycocalyx causes endothelial dysfunction in FD and is partially reversible by FD-specific treatment. Future studies are now warranted to analyze whether FD-specific treated patients will additionally profit by a general anti-inflammatory therapy and/or by a more target-specific protective Tie2 treatment of the glycocalyx.

## Limitations

Our cell culture experiments demonstrated a relative fast recover of the endothelial glycocalyx after treatment with FD-specific therapies. It is possible that the treatment effect is greater in the migalastat-treated group because the overall duration of Fabry-specific therapy was shorter in these patients and the main change under therapy may be more visible at the beginning. Thus, from the current setup, no conclusions should be drawn on the effectiveness of either of the two therapies on glycocalyx recovery in FD. The longer the therapy, the less obvious are therapeutic changes in the course. Although we identified inflammation and glycocalyx degeneration as early processes in our study, it cannot be excluded that additional pathophysiological pathways are involved in the endothelial dysfunction in FD patients, too. None of the patients was treated by specific anti-inflammatory (including Tie2 inhibitors) or anti-oxidative medication. However, one patient receiving migalastat and two patients under ERT were on immune-suppressive medication due to cardiac and kidney transplantations, respectively. The lack of a control group without variants within the *GLA* gene might be a limitation of this study.

## Data Availability Statement

The original contributions presented in the study are included in the article/**Supplementary Material**. Further inquiries can be directed to the corresponding author.

## Ethics Statement

The studies involving human participants were reviewed and approved by Medical Association of Westphalia-Lippe and the Ethical Committee of the Medical Faculty of the University of Muenster (project-nos.: 2011-347-f; 2011-186-f). The patients/participants provided their written informed consent to participate in this study.

## Authors Contributions

ML and EB designed the concept and methodology. ML, SP, DS, and DM conducted the experiments. ML and EB wrote the paper. ML, DM, and EB provided resources. All authors analyzed and interpreted the data. All authors contributed to the article and approved the submitted version.

## Funding

Parts of this work were supported by the funds ‘Innovative Medical Research’ of the University of Muenster Medical School (LE221801), the German Research Foundation (DFG) grant MA8629/1-3, and by an Investigator-Initiated Research grant from Takeda (no. IIR-DEU-001323). Inflammatory marker analyses were supported by Amicus Therapeutics, which is gratefully acknowledged. The technical support of IEM and GlycoCheck and Microvascular Health Solutions is also gratefully acknowledged. The funders were not involved in the study design, collection, analysis, interpretation of data, the writing of this article or the decision to submit it for publication.

## Conflict of Interest

ML received speaker honoraria, travel funding and research grants from Sanofi Genzyme, Shire Corporation/Takeda, and Amicus Therapeutics. EB received research grants and speaker honoraria from Sanofi Genzyme, Shire Corporation/Takeda, Amicus Therapeutics, and Greenovation/Eleva.

The remaining authors declare that the research was conducted in the absence of any commercial or financial relationships that could be construed as a potential conflict of interest.

## Publisher’s Note

All claims expressed in this article are solely those of the authors and do not necessarily represent those of their affiliated organizations, or those of the publisher, the editors and the reviewers. Any product that may be evaluated in this article, or claim that may be made by its manufacturer, is not guaranteed or endorsed by the publisher.
