# Supplementary material for: Endothelial Dysfunction in Fabry Disease Is Related to Glycocalyx Degradation
Source: Front Immunol. 2021 Nov 30;12:789142. doi: 10.3389/fimmu.2021.789142 (PMC8670230; doi:10.3389/fimmu.2021.789142)
Supplement: Supplementary file 1 [file DataSheet_1.pdf]

Supplementary Material

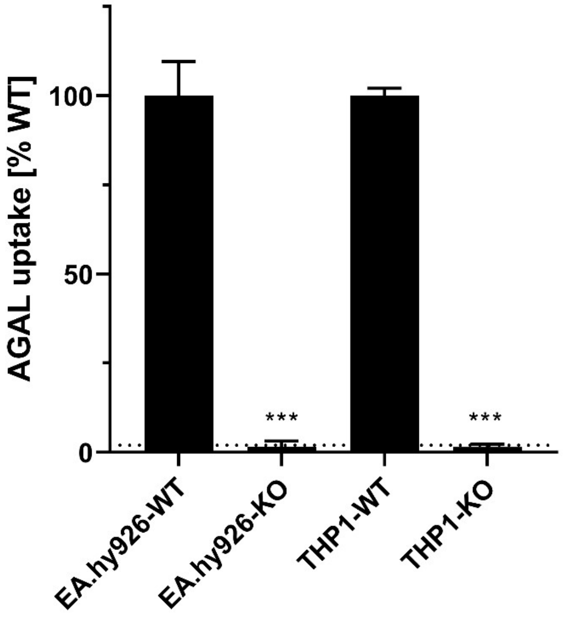

Supplemental Figure 1: α-Galactosidase A activities in wild-type EA.hy926 and THP1 monocytes compared to CRISPR/Cas9-mediated AGAL-knockouts. The dashed line marks 2% residual AGAL activity of wild-type. ***p<0.001 determined by unpaired two-tailed Student's T test.

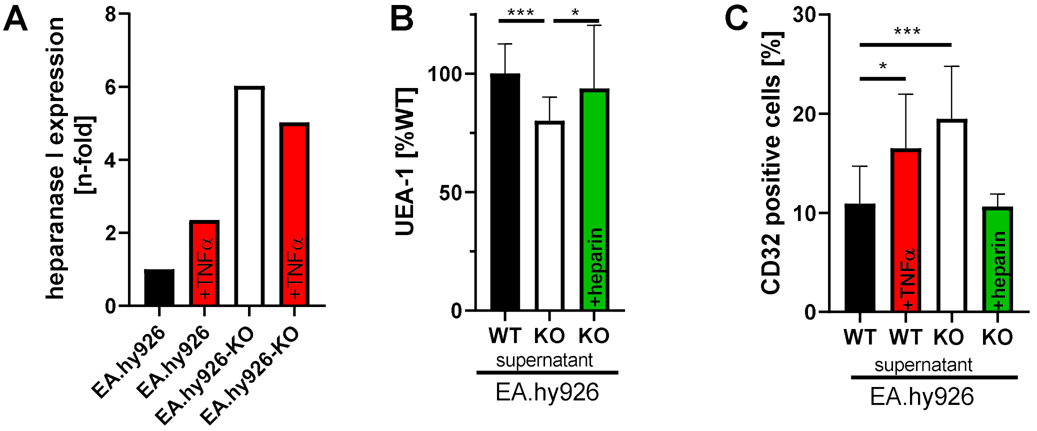

Supplemental Figure 2: Heparanase I expression is increased in AGAL-deficient endothelial cells and cell culture supernatants from AGAL-deficient endothelial cells degrade the glycocalyx of wild-type cells. A) AGAL-deficient EA.hy926 (EA.hy926-KO) cells showed an increased heparanase 1 expression compared to wild-type (WT). **B)** L-fucose (UEA-1) staining of EA.hy926 cells treated with supernatants of WT or AGAL-deficient cells (KO) for 30 minutes confirmed that soluble components released by the KO cells mediated glycocalyx reduction. Glycocalyx degradation activity was inhibited by heparin treatment. **C)** THP1 (WT) adhesion on EA.hy926 (WT) cells with supernatants of wild-type (WT) or AGAL-deficient cells (KO) for 30 minutes. Effects of KO supernatants were reversible by pre-treatment of the media with 0.4 U/ml heparin for 10 minutes. *$p<0.05$, ***$p<0.001$ determined by One-Way ANOVA.

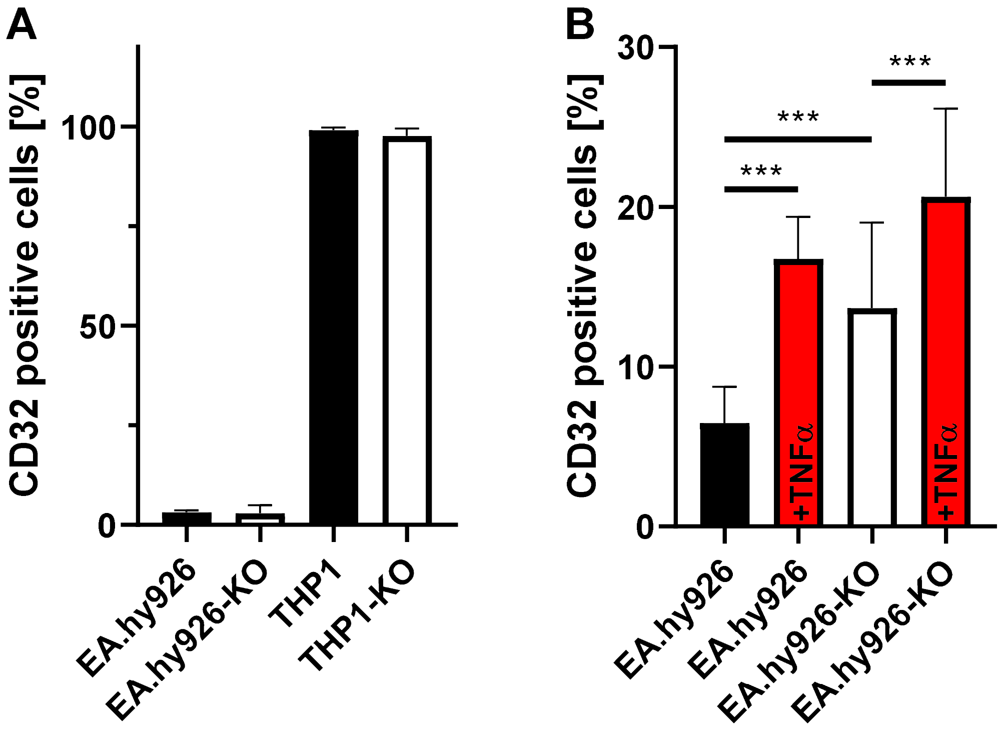

Supplemental Figure 3: THP1 monocyte adhesion on endothelial EA.hy926 cells. A) Only THP1 monocytes were positive for CD32 and AGAL-deficiency had no effect on CD32 expression. **B)** Monocyte adhesion was significantly higher in an AGAL-deficient background compared to wild-type. TNFα increased monocyte adhesion in a wild-type and AGAL-deficient background. ***p<0.001 determined by One-Way ANOVA.

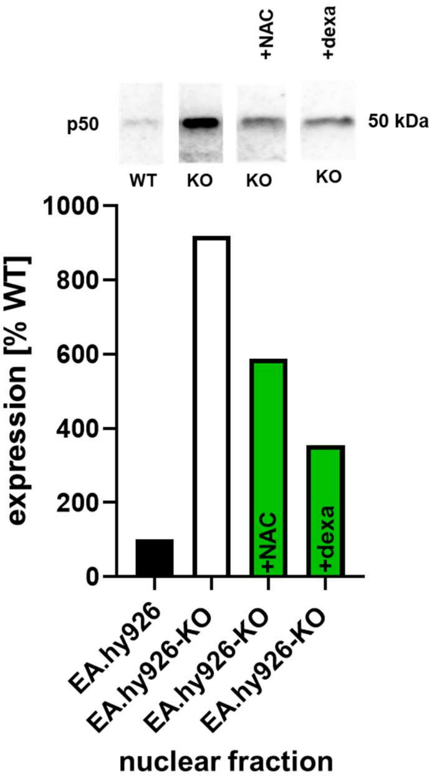

Supplemental Figure 4: **Increased NF-κB signaling in AGAL-deficient cells.** AGAL-deficient EAh.hy926 cells showed increased nuclear NF-κB (p50 subunit) localization, which could be reduced by dexamethasone (dexa, 60 µM) and N-acetylcysteine (NAC, 20 nM) treatment for 24 h. Representative western blot and analysis from N=4 independent experiments.
